# Editorial Special Issue TGF-Beta/BMP Signaling Pathway

**DOI:** 10.3390/cells9112363

**Published:** 2020-10-27

**Authors:** Isabel Fabregat, Blanca Herrera, Aránzazu Sánchez

**Affiliations:** 1 TGF-β and Cancer Group, Oncobell Program, Bellvitge Biomedical Research Institute, (IDIBELL) and University of Barcelona, L’Hospitalet de Llobregat, 08908 Barcelona, Spain; 2 Oncology Program, CIBEREHD, National Biomedical Research Institute on Liver and Gastrointestinal Diseases, Instituto de Salud Carlos III, 28029 Madrid, Spain; 3 Department of Biochemistry and Molecular Biology, Faculty of Pharmacy, Complutense University of Madrid (UCM), Health Research Institute of the Hospital Clínico San Carlos (IdISSC), 28040 Madrid, Spain

The transforming growth factor β (TGF-β) superfamily plays key roles in development and tissue homeostasis, controlling the maintenance and regeneration of mature tissues. Cytokines belonging to this family can be multifunctional (TGF-β and bone morphogenetic proteins, BMPs) or develop highly specialized functions (anti-Müllerian hormone, AMH, or growth differentiation factor 8, myostatin, GDF8) and they control a variety of cellular processes such as proliferation, differentiation, cell death, adhesion and movement, metabolism, pluripotency and stemness.

This special issue comprises 27 papers, including 18 original articles and 9 reviews, which cover a broad range of topics including cancer, fibrosis, wound healing, neuroinflammation, stem cell biology, organogenesis and tissue homeostasis, in which TGF-β family signaling plays an active and important role, highlighting once more the remarkable widespread actions of the TGF-β and BMP ligands. 

Several works included in this special issue focus on the study of different steps or components of TGF-β signaling pathways, in a cellular- and context-dependent manner. Endoglin (ENG), a TGF-β auxiliary receptor, is the focus of two studies. Gallardo-Vara et al. [1] describe novel partners for soluble endoglin (sENG), a circulating form of ENG that is a proteolytically released fragment of the membrane-bound protein. More than 20 new sENG interacting proteins are identified, and two of them are further studied: galectin 3, a secreted member of the lectin family, involved in the pathogenesis of many human diseases, including pathological angiogenesis and endothelial dysfunction; and tripartite motif-containing protein 21 (TRIM21), a E3 ubiquitin ligase belonging to the TRIM family that could be involved in the ubiquitinization and degradation of ENG. sENG is involved in cardiovascular, inflammatory and tumor pathologies, and it is known to have an antiangiogenic function, but it is still a largely unknown protein. Clearly, the study of a sENG interacting protein network opens new research paths to better understand ENG function in different pathophysiological contexts and the relevance for TGF-β and BMP signaling in such scenarios. Meurer et al. [2] investigate the membranal subdomain localization of ENG in hepatic stellate cells, a cell population at the cellular epicenter of the hepatic fibrotic process. Authors demonstrate that ENG mostly localizes in the lipid rafts, and more importantly, they evidence the presence of ENG in hepatic stellate cell-derived exosomes. A role for ENG in the regulation of the fibrogenic process by altering TGF-β signaling has been pointed out before, but these new findings suggest a specific role for this signaling molecule as part of the exosome-mediated intercellular communication between different hepatic cell populations, a process which is believed to play a key role during pathogenesis of different liver diseases, including liver fibrosis. 

TGF-β signaling is tightly controlled by numerous positive and negative feedback loops, with some of these regulatory circuits being miRNA dependent. Yang et al. [3] describe a novel regulatory axis operating in porcine granulosa cells involving miR1306/TGFBR2/SMAD4. Authors propose a mechanism that seems to act as a reciprocal regulatory system, where miR1306 negatively regulates TGF-β signaling by targeting TGFBR2 while Smad4 negatively regulates miR1306 transcription. Interestingly, miR1306 expression is increased during follicular atresia of porcine ovaries and promotes granulosa cell apoptosis by inhibiting TGF-β signaling, providing new clues on the participation of TGF-β signaling in this physiological process. A related work by Ding et al. [4] focuses on miR-202-5p, a miRNA also targeting TGFR2, in follicle growth. miR-202-5p is expressed in granulosa cells of goat growing follicles and participates in another negative feedback loop between steroidogenic factor (SF1) and TGF-β signaling, where SF1 binds to miR-202 promoter and upregulates its expression, that in turn targets TGFR2, resulting in attenuation of SMAD signaling and granulosa cell apoptosis.

As pointed out before, depending on the cell type, TGF-β family members regulate a large number of cellular responses. Dysregulation of the TGF-β superfamily signaling is implicated in various human diseases including cancer, fibrosis, autoimmune diseases and vascular disorders, although the pathological mechanisms are not completely understood. Special efforts are being placed on deciphering their mode of action in tissue fibrosis and cancer either to define novel and specific targets of intervention or to provide sound basis for treatment/patient selection. Several papers in this special issue move in this direction. Valencia et al. [5] reveal a mechanism contributing to immune evasion of acute lymphoblastic leukemia (ALL) cells, in which BMP4 is a key player. Using different targeted strategies, authors show that BMP4 is produced by ALL cells, and it impairs dendritic cell and macrophage differentiation. As a result, dendritic cells acquire immunosuppressive features and macrophages are pushed to a M2-like phenotype, in both cases implicating enhanced protumoral features in the cells. More work is needed to determine whether blockade of BMP4 secretion by ALL cells could contribute to control leukemia progression by acting on the microenvironment. Related to the immunosuppressive actions of TGF-β, Sow et al. [6] explore whether the antitumor effect of PD-L1 blocking antibody could be enhanced by pharmacological inhibition of TGF-β, by using two tumor models, highly immunogenic (MC38 colon adenocarcinoma) and poorly immunogenic (KPC1 pancreatic tumor), both producing high levels of TGF-β1. Only highly immunogenic tumor cells showed improvement with combined treatment, highlighting that tumor immunogenicity is a dominant feature predicting responsiveness to dual targeting TGF-β signaling and PD-L1. This work is evidence of the importance of the right selection of tumors for an efficient response to combinatorial immunotherapy regimes. 

Another study, from Sundqvist et al. [7], explores the role of JNK-dependent cJun phosphorylation in TGF-β pro-oncogenic response in pre-malignant MCF10A-RAS breast cancer cells. Authors show that JNK signaling can exert negative regulatory effects on tumor cell migration and invasion; that is, it exerts anti-oncogenic functions, interfering with the SMAD-AP-1 regulated epithelial–mesenchymal transition (EMT) process via cJun phosphorylation and altering the expression of specific Jun/Fos target genes. Defining molecular pathways specifically controlling certain TGF-β functions is relevant because despite TGF-β being a target for antitumor therapies, systemic application of TGF-β inhibitors may result in important side effects, so it is clear that more specific interventions are required. Identification of downstream and more delimited target molecules may allow the development of more personalized and safer therapeutic strategies.

Besides cancer, this special issue includes a number of works focused on other diseases in which TGF-β family ligands are important. Hemorrhagic hereditary telangiectasia (HHT) is a disorder that results in the development of abnormalities in blood vessels, which presents with two forms: HHT1, characterized by pulmonary and cerebral arteriovenous malformations, caused by mutations in the ENG gene, and HHT2, which is associated with vascular hepatic malformations and is caused by mutations in ALK1 (ACVRL1 gene), a TGF-β type I receptor with high affinity for BMP9. Using valuable human telangiectasia biopsies from HHT1 patients, Iriarte et al. [8] show an increased activation of the AKT pathway that could be associated with endothelial cell proliferation, a key step in the pathogenic process of vascular malformations. These results add to evidence in the literature that associates PI3K activation with arterial venous malformations and proposes the AKT pathway as a target for HHT treatment. Caruso et al. [9] show that TGF-β is involved in the mechanism of action of carnosine, an endogenous antioxidant, against neurodegeneration and disease progression in Alzheimer´s disease using different in vitro approaches. In fact, this evidence supports that the rescue of TGF-β signaling is an interesting strategy for protecting against inflammation and neurodegeneration in Alzheimer´s disease. Pulmonary arterial hypertension (PAH) is strongly linked to alterations in the BMPR2 signaling pathway. Mutations in the BMPR2 gene are found in hereditary PAH, and aberrant BMPR2 signaling is behind the development of non-hereditary PAH. Happé et al. [10] describe that animal models used for the study of PAH (the monocrotaline and the Sugen–Hypoxia models) only partially recapitulate the human disease in terms of the BMPR2 pathway, highlighting the need for appropriate disease models for a deeper understanding of the mechanisms driving pathology and to support translational research.

In the last years, BMP9 study has gathered much attention, with special interest on its role in liver physiology and pathophysiology. Here, two research groups provide new information about the mechanisms of BMP9 action in liver pathology. In an elegant work, Gaitanzti et al. [11] provide new mechanisms by which BMP9 acts as a driver of liver fibrogenesis. BMP9 potentiates lipopolysaccharide (LPS)-driven capillarization in liver sinusoidal endothelial cells (LSEC) and macrophages. Furthermore, BMP9 and LPS synergistically induce the expression of pro-inflammatory cytokines, data that point out the BMP9 contribution to the inflammation process that is associated with liver fibrosis. On the other hand, Desroches-Caster et al. [12] extend a previous work where they showed that genetic deletion of BMP9 in 129/Ola mice led to a profound alteration of LSEC and spontaneous hepatic perisinusoidal fibrosis [13], further supporting a key role for BMP9 in liver homeostasis, a role supported by our own previous data as well [14,15]. In this new article, they prove that the hepatic alterations provoked by BMP9 deletion are mouse strain-dependent. Thus, different to the 129/Ola mouse strain, the C57/Bl6 mouse strain did not develop LSEC capillarization and the associated liver fibrosis. Authors also provide evidence of different life expectancies associated with BMP9 deletion in different mouse strains (C57/Bl6, 129/Ola and BALB7C). This work proposes the interesting idea that the key function of BMP9 in liver homeostasis is dependent on genetic modifiers that are far from being understood. 

Kovermann et al. [16] cover another topic, the role of TGF-β and BMP ligands in chondrogenesis and cartilage repair and its application in regenerative medicine. Synovial-derived stem cells (SDSCs) are a promising cell source for tissue engineering applications in cartilage repair due to the simplicity of isolation and natural location within the articular joint, plus evidence of their multilineage differentiation potential particularly toward the chondrogenic lineage. In this work, authors examine the influence of TGF-β, BMP2 and dexamethasone on SDSCs chondrogenesis in vitro. Using human SDSCs isolated from the synovial membrane, authors induced chondrogenic differentiation in 3D pellet culture with a chondrogenic medium including various concentrations of TGF-β1 and BMP2, with or without dexamethasone. Removal of dexamethasone improved chondrogenesis by leading to a more stable chondrogenic phenotype and a less hypertrophic-like (osteogenic) phenotype, which is considered beneficial for resting cartilage. This information might prove useful for applications in the field of regenerative medicine, particularly for patients with severe cartilaginous problems.

TGF-β signaling talks and crosstalks with many different signaling pathways, and while some of them have been known for a number of years, the landscape is far from being complete, as these crosstalks are dependent on the cell type and cellular context. Indeed, a lot of effort is aimed at unraveling these TGF-β networks, because of their importance in providing specific and contextualized biological responses determinant for the outcome of physiological and pathological processes. Not surprisingly, this research area is also well represented in this special issue.

Bernatik et al. [17] explore the mechanism behind the crosstalk between TGF-β/BMP and Wnt pathways. Authors show how Smurf2 (an E3 ubiquitin ligase acting as a negative regulator of TGF-β/BMP signaling) influences DVL function, a key cytoplasmic signal transducer of the Wnt pathway. Results show that DVL activates Smurf2 that in turn allows Smurf2 to efficiently ubiquitinate substrates not only of Wnt/PCP (including DVL and Prickle1) but of TGF-β/BMP pathways. Interestingly, the mechanism for DVL effect has also been revealed, so that the ability of DVL to increase Smurf2 ubiquitin activity is associated with release of Smurf2 autoinhibition and involves DVL DIX domain and Smurf2 HECT domain. These results highlight that DVL has the potential to regulate TGF-β/BMP signaling and such an effect is fully dependent on Smurf2 activity, unraveling a novel interconnection mechanism between Wnt and TGF-β/BMP pathways.

Yin et al. [18] show a crosstalk between TGF-β and glial cell line derived neurotrophic factor (GDNF), a distant member of TGF-β superfamily, in the control of follicular function. In this work, authors use in vitro genetic and pharmacological approaches using human granulosa cells to provide a signaling mechanism by which TGF-β1 regulates the follicular function. Results show that TGF-β induces the production, maturation (a process involving Furin pro-protein convertase) and secretion of GDNF, a factor known to be involved in the control of follicular activation and oocyte maturation. Specific components of the TGF-β signaling pathway involved are identified. 

A crosstalk between nitric oxide (NO) and BMP2 signaling during osteogenesis is shown by Differ et al. [19] using different in vitro pharmacological approaches. The NO pathway increases BMP2 signaling via protein kinase A activation and leads to improved BMP2 osteogenic activity in the myoblast cell line C2C12. Awaiting in vivo experiments to confirm these in vitro observations, these data could be of interest in the clinic, particularly for the treatment of open tibia fractures, where there is a need to reduce BMP2 dose in the clinical practice to diminish high-dose-induced side effects and enhance BMP2 therapeutic efficiency.

Finally, work from our laboratory included in this special issue [20] unmasks a new crosstalk involving BMP9 and the hepatocyte growth factor (HGF) in adult liver progenitor cells, a cell population with an important role in liver regenerative responses upon chronic damage. We demonstrate an interconnection between HGF and BMP9 pathways via the ALK1/SMAD1 signaling axis that modulates cell response. Interestingly, HGF/c-Met signaling is able to block BMP9-mediated cell apoptosis by shifting BMP9-triggered signaling, potentiating SMAD1 activation (via ALK1) and weakening the activation of the non-canonical p38MAPK, which mediates the apoptotic response. Our work not only illustrates the complexity of the signaling network established during chronic liver injury but contributes to enhance our knowledge on BMP9 mechanisms of action in hepatic cells while revealing novel mechanisms responsible for the known protective activity of HGF/c-Met signaling. A deep knowledge of the signaling mechanisms of key hepatic regulators is essential to design effective therapeutic interventions to reduce or revert liver damage in patients suffering from chronic liver diseases.

Together with these original research manuscripts, a few stimulating review manuscripts on various topics are included in this special issue, addressing pathological roles of TGF-β/BMP signaling in fibrosis and cancer [21,22,23,24,25], their role in physiological conditions [26] or both [27]. A deep look into BMP signaling mechanisms is also provided by Nickel and Muller [28].

It is clear that a lot of research is going on in relation to the TGF-β/BMP signaling in very different research areas, all of them equally exciting and important. We are happy to have been able to put together in this issue some of those research activities showing us the advances in this area, with much more to come. 
